# Developing and evaluating an interprofessional shared decision-making care model for patients with perinatal depression in maternal care in urban China: a study protocol

**DOI:** 10.1186/s12875-023-02179-2

**Published:** 2023-11-03

**Authors:** Defang Xiang, Xian Xia, Di Liang

**Affiliations:** 1 School of Public Health, National Health Commission Key Laboratory of Health Technology Assessment, Fudan University, Shanghai, China; 2 Obstetrics and Gynecology Hospital of Fudan University, Shanghai, China

**Keywords:** Perinatal depression, Shared decision-making, Decision aid, Participatory design, Qualitative methods, RCT, Economic evaluation

## Abstract

**Background:**

The majority of patients with perinatal depression (PND) in China do not receive adequate treatment. As forming a therapeutic alliance with patients is crucial for depression treatment, shared decision-making (SDM) shows promise in promoting patients’ uptake of evidence-based mental health services, but its impact on patient outcomes and implementation in real-world maternal care remain uncertain. Therefore, this study aims to develop and evaluate an interprofessional shared decision-making (IP-SDM) model for PND to enhance maternal mental health services.

**Methods:**

This study contains four research phases: feasibility testing (Phase 1), toolkit development (Phase 2), usability evaluation (Phase 3), and effectiveness evaluation (Phase 4). During the development stage, focus group interviews will be conducted with expectant and new mothers, as well as maternal care providers for feasibility testing. A toolkit, including a patient decision aid along with its user guide and training materials, will be developed based on the findings of Phase 1 and syntheses of up-to-date evidence and appraised by the Delphi method. Additionally, a cognitive task analysis will be used for assessing the usability of the toolkit. During the evaluation stage, a prospective randomized controlled trial embedded in a mixed methods design will be used to evaluate the effectiveness and cost-effectiveness of the IP-SDM care model. The study targets to recruit 410 expectant and new mothers who screen positive for depression. They will be randomly assigned to either an intervention group or a control group in a 1:1 ratio. Participants in the intervention group will receive decision aid, decision coaching, and clinical consultation, in addition to usual services, while the control group will receive usual services. The primary outcome is the quality of decision-making process, and the secondary outcomes include SDM, mental health service utilization and costs, depressive symptoms, and health-related quality of life. In-depth interviews will be used to explore the facilitating and hindering factors of SDM.

**Discussion:**

This study will develop an IP-SDM care model for PND that can be implemented in maternal care settings in China. This study will contribute to the understanding of how SDM impacts mental health outcomes and facilitate the integration of mental health services into maternal care.

**Trial registration:**

ChiCTR2300072559. Registered on 16 June 2023.

**Supplementary Information:**

The online version contains supplementary material available at 10.1186/s12875-023-02179-2.

## Contributions to the literature

This study aims to develop a culturally relevant and responsive IP-SDM care model for PND that can be implemented in maternal care settings in China. For instance, the IP-SDM model will involve family members in the decision-making process between patients and providers.

This study will contribute to the understanding of how SDM impacts mental health outcomes. This study will not only investigate the effects of SDM on mental health outcomes but also explore the potential mechanisms underlying such impacts, using causal mediation analysis.

Lastly, this study will conduct a randomized controlled trial design to evaluate the effectiveness and cost-effectiveness of the care model, providing evidence for implementing SDM in clinical practice within the context of integrated mental health services.

**Supplementary Information:**

The online version contains supplementary material available at 10.1186/s12875-023-02179-2.

## Background

Perinatal depression (PND) is a major depressive episode that occurs during pregnancy or within 4 weeks after delivery [1]. According to recent systematic reviews and meta-analyses, the prevalence of PND is 11.9% worldwide, while the prevalence of PND in China is as high as 16.3%, including 19.7% for prenatal depression and 14.8% for postnatal depression [2]. Clinical guidelines often recommend structured psychotherapy for mild and moderate perinatal depression and antidepressants along with referral to a psychiatrist for those with severe perinatal depression [3, 4]. Untreated perinatal depression not only places a heavy burden of disease on women but may also affect the long-term health and development of children [5, 6].

To date, the vast majority of patients suffering from PND have not received adequate treatment. Recent systematic reviews and meta-analyses have shown that even with universal screening, only 43% of women screened for possible perinatal depression received further diagnostic evaluation or intervention [7]. China still lacks data on the healthcare utilization pattern among patients with perinatal depression. However, given only 0.5% of patients with depression in the general population receive adequate treatment [8], the proportion of patients who have received adequate treatment for perinatal depression is likely to be low. One study even found [9] that in a cohort of 1126 women screened for perinatal depression, 22% were likely to have it, but only three expressed interest in the free psychiatric care offered, and only one accepted the care.

Interactions and relationships between patients and healthcare providers play a crucial role in the treatment of common mental disorders, such as depression. The extent to which patients feel heard, respected, and trusted by their healthcare providers will directly impact whether or not they accept the recommended treatment [10]. Also, using treatment modalities that correspond to the patient’s preferences has a significant impact on achieving positive treatment outcomes [11, 12]. Therefore, taking patients’ needs and preferences into account and forming a therapeutic alliance with them is the starting point and foundation of depression treatment [13, 14].

Shared decision-making (SDM) is an important approach and best practice in establishing a strong therapeutic alliance between patients and healthcare providers in clinical decision-making [15, 16]. Based on the best available evidence [17, 18], SDM is characterized by the involvement of at least two parties (typically the physician and the patient), sharing of information, gradual reaching of consensus on the preferred choice, and making a concerted decision together [19, 20]. SDM encourages patients to think about the benefits, risks, and uncertainties of different options, communicate their needs and preferences, and make the most advantageous clinical decisions with their doctors. SDM is particularly appropriate in situations that require long-term patient-physician interaction and where different intervention options have their advantages and disadvantages [21].

During pregnancy, there are advantages and disadvantages to psychotherapy, pharmacotherapy, and a combination of the two treatments for depression [22], and mothers may have individual treatment needs and preferences, such as some mothers may wish to continue follow-up without therapy for a while [23, 24]. The current evidence-based medicine evidence is insufficient to support physicians in predicting which treatment modality will work best for a specific patient [25]. Therefore, doctors particularly need to make decisions based on maternal needs and preferences, and carefully weigh the potential benefits and risks of different options with the mother.

While decision aid and decision coaching are effective ways to provide decision support to patients and promote SDM, studies related to SDM in mental health are primarily based on populations from high-income countries. Evidence shows that using a decision aid in the treatment of PND is feasible and acceptable, and may reduce decisional conflict [26, 27]. However, further research is warranted to examine its impact on mental health service utilization and depression outcomes [28, 29] and its implementation in primary care settings. There is still a lack of decision aid for perinatal depression as well as an SDM model involving multiple stakeholders for the Chinese population. Moreover, as the impact of SDM on healthcare resource consumption remains controversial, it is warranted to conduct an economic evaluation to assess the cost-effectiveness along with the effectiveness of implementing SDM for perinatal depression in China’s healthcare system.

This study aims to design a decision aid tailored to Chinese patients with perinatal depression. The decision aid will serve as the centerpiece for developing a novel shared decision-making model that integrates multiple parties, such as multi-specialty and multi-professional medical personnel, patients, and their families. The study hypothesizes that this model would have a positive impact on the quality of the decision-making process, mental health service utilization, and mental health outcomes.

### Research ethics

The study was approved by the Ethics Committee, School of Public Health, Fudan University (IRB# 2023-04-1034) and registered at https://www.chictr.org.cn/. In this study, each participant will be required to sign an informed consent form.

## Methods

### Conceptual framework

**Fig. 1:**
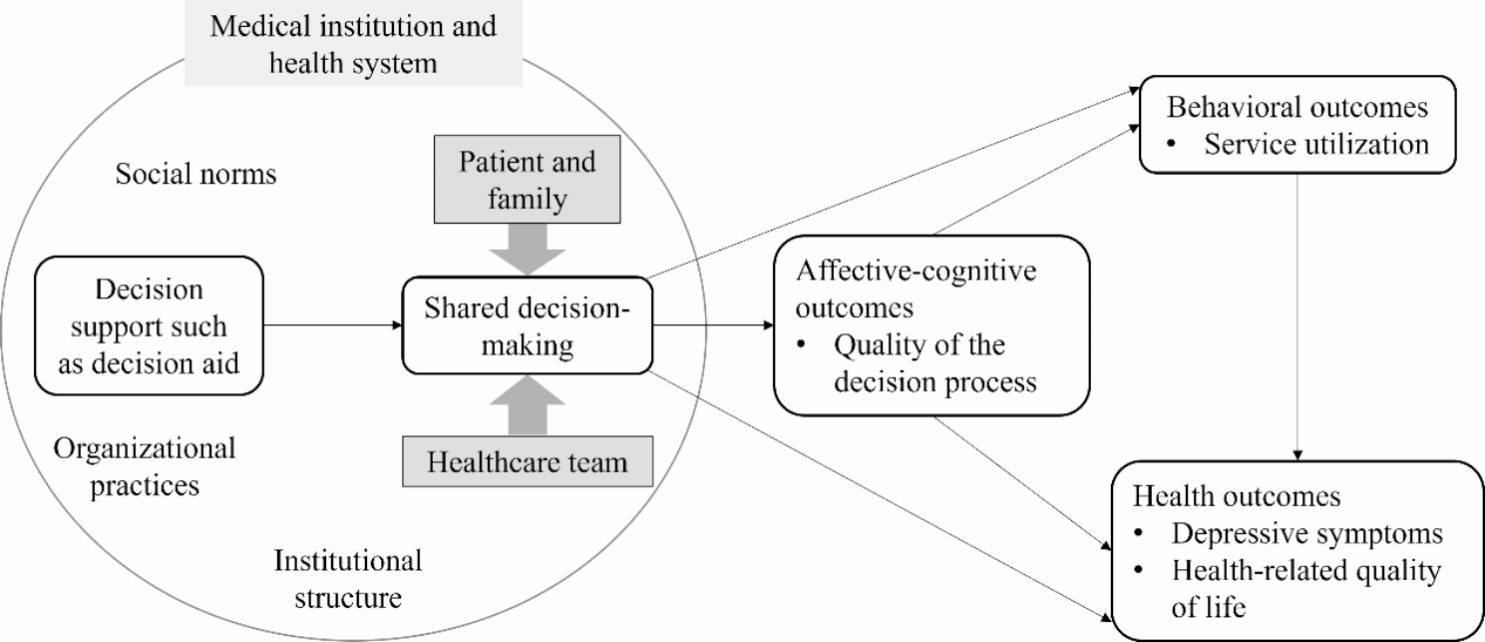
Theoretical framework of this study

This study will be guided by a conceptual framework which integrates Shay’s conceptual framework of SDM affecting patient outcomes and the interprofessional shared decision-making model (IP-SDM) (Fig. 1). Shay’s conceptual framework proposes that SDM may have direct effects on patient affective-cognitive outcomes, behavioral outcomes, and health outcomes [30]. The IP-SDM expands the perspective of SDM beyond the patient-practitioner dyad to take healthcare teams and broader policies and social context into account [31, 32].

The conceptual framework of this study suggests that providing patients with decision support, such as decision aid, will promote the implementation of SDM. This, in turn, may influence patient affective-cognitive outcomes (e.g., quality of the decision process), behavioral outcomes (e.g., service utilization), and health outcomes (e.g., depressive symptoms, health-related quality of life). SDM may affect patients’ health outcomes directly or indirectly through affective-cognitive and behavioral outcomes. Additionally, whether SDM can be achieved is also influenced by various factors at different levels, including the patient and family, healthcare team, medical institution, and health system. These factors may include social norms, institutional structure, organizational practices, and so on.

### Study overview

This study consists of four research phases: feasibility testing (Phase 1); toolkit development (Phase 2); usability evaluation (Phase 3); effectiveness evaluation (Phase 4). Through the development stage (Phase 1 to Phase 3), the toolkit will be refined iteratively based on the findings of each phase. In the evaluation stage (Phase 4), an RCT will be conducted to evaluate the effectiveness of the toolkit.

A participatory design approach will be adopted to ensure that the toolkit developed meets the needs of key stakeholders, particularly expectant and new mothers. To adopt the participatory design approach, this study will establish an advisory committee comprising 2–3 women with a history of perinatal depression and their family members, as well as maternal care and mental health providers. The committee will work together to finalize the design of a patient decision aid for perinatal depression along with any related interventions. The research team will work with the advisory committee through multiple rounds of consultation sessions. Furthermore, the feedback and comments of users and providers will be incorporated into the design of the patient decision aid and related interventions through focus group interviews, the Delphi method, and cognitive task analysis.

This study complies with the Standard Protocol Items: Recommendations for Interventional Trials (SPIRIT) (Supplementary file 1).

### Development stage

#### Phase 1. Feasibility testing

This phase will consist of assessing the feasibility of implementing the patient decision aid for perinatal depression and SDM by focus group interviews with expectant and new mothers and maternal care providers. The research team will recruit expectant and new mothers as well as maternal care providers from selected study sites including various maternal and child health institutions and general hospitals in Shanghai. Each interview participant will be required to sign an informed consent form.

The inclusion criteria for interviewees require individuals to be 18 years of age or older. Expectant and new mothers should register at selected study sites to receive maternal health services, and maternal care providers should be involved in maternal health-related work at these sites.

The exclusion criteria for interviewees include individuals ever diagnosed with serious mental illness, namely schizophrenia, delusional disorder, schizoaffective disorder, bipolar disorder, psychotic disorder due to epilepsy, and mental retardation with psychotic symptoms. Additionally, individuals who have other physical illness or mental disorders that require immediate inpatient treatment will also be excluded.

Focus group interviews will be conducted with 5–8 members per group to balance the amount of information in each group and the opportunity for each participant to share. At least three separate focus group interviews will be conducted among expectant and new mothers, as well as maternal care providers, respectively. However, the final number of groups will be determined according to data saturation. The interviews will primarily cover the perceived decisional needs of expectant and new mothers, such as the information needed and priorities. Additionally, participants will provide their views on the strengths and weaknesses of the patient decision aid developed in this study, as well as areas for improvement.

All audio recordings of the interviews will be transcribed verbatim at the end of the interview. The analysis of the interview scripts will be based on the principles of grounded theory, using NVIVO 12 (Lumivero, Denver, Colorado, USA). Two members of the research team will independently read the interview transcripts and inductively code them using open coding in order to form a coding system. The main themes that emerge will be subsequently identified.

#### Phase 2. Toolkit development

The toolkit will include the patient decision aid for PND along with its user guide and training materials. Based on IP-SDM and the findings of Phase 1, the patient decision aid will be developed according to the International Patient Decision Aids Standards (IPDAS) criteria [33] which provides explicit guidance on the content, development process, and effectiveness of patient decision aids. The patient decision aid will be developed for expectant and new mothers who have screened positive for PND. Its content will include: eliciting the patient’s general priorities of care; eliciting his/her specific goals and outcomes related to perinatal depression; outlining various treatment modalities (e.g., psychotherapy, pharmacotherapy, follow-up without treatment) for PND and detailing population-specific benefits and risks. The initial toolkit will be Chinese language and should use concise language to ensure easy understanding by patients.

The research team prepares initial statements regarding treatment modalities and correspondent benefits and risks based on published literature. The retrieved literature was categorized according to an evidence hierarchy. The highest available levels of evidence, such as systematic reviews and meta-analyses, were prioritized for statement development. If no systematic reviews are available, randomized controlled trials and observational studies will provide evidentiary support. The statements will be refined through an iterative process with the advisory committee. The finalized statements will be incorporated into the toolkit. After being approved by the advisory committee, the toolkit will be distributed to Delphi panelists.

The Delphi method will be used to evaluate the toolkit. The Delphi method is a robust method to identify the collective opinion of experts. This study will recruit at least 20 domestic and international experts as Delphi panelists. The experts will be selected on multiple years of clinical and/or research experience with maternal health and mental health. The list of experts will be generated by tracking publications, conferences, national/international networks, and suggestions from contacted experts. The experts will be consulted in two to three anonymous Delphi rounds until a consensus is reached. During each round, experts will rate the accuracy, comprehensiveness, balance of perspective, and ease of understanding of the toolkit based on their expertise. After the Delphi process, the toolkit will be further revised on the feedback.

#### Phase 3. Usability evaluation

A cognitive task analysis (CTA) will be used for assessing the usability of the toolkit for facilitating SDM in this study. CTA is a family of psychological research methods that focuses on the cognitive components of effective task performance, aiming at eliciting the knowledge needed to perform a task in order to improve task performance. Previous studies have shown that 80% of usability problems can be found in 5–8 participants [34]. This study will conduct three rounds of CTA, and each round will recruit four to five pairs of maternal care providers and expectant or new mothers. The inclusion and exclusion criteria for participants are consistent with the focus group interviews, with the exception that expectant or new mothers should have an Edinburgh Postnatal Depression Scale (EPDS) score ≥ 13 during pregnancy or within 6 weeks postpartum. All healthcare professionals in the study who will provide interventions in the subsequent randomized controlled trial are required to participate in the cognitive task analysis.

Each CTA interview will last approximately one hour. Before the interview, expectant or new mothers will be provided with the decision aid for PND, and maternal care providers will be trained to use the toolkit. During the interview, the research team will first introduce the task that participants need to complete: accomplishing SDM following the steps of IP-SDM using the decision aid. The research team will observe the interaction between mothers and professionals and take notes of participants’ use of the decision aid, including any confusion, errors, and the time spent using the tool. The research team will also evaluate the extent of SDM using the Observing Patient Involvement in Decision Making (OPTION) scale which was developed by Elwyn et al. [35] to measure the extent to which physicians facilitate patient involvement in decision-making from a third-party, observer perspective. In addition, all participants will be asked to “think aloud” as they perform the task with the toolkit. After completing the task, participants will be interviewed immediately about their perceptions (e.g., strengths and weaknesses) of the decision aid and its instructions and training materials, as well as the extent to which the toolkit supports the SDM process. After the interview, the interview data will be analyzed using the same methods as the focus group interviews.

The toolkit will be revised iteratively according to the feedback of each round of CTA. In the final round, the mean score for each item on the final OPTION scale should be greater than or equal to 2 [36].

### Evaluation stage

This study will conduct a randomized controlled trial (RCT) embedded in a mixed methods design.

### Quantitative evaluation

This study will use an RCT design to evaluate the effectiveness and cost-effectiveness of the IP-SDM care model for perinatal depression.

### ① Study site

This study will select the Obstetrics & Gynecology Hospital of Fudan University as the study site for the RCT. The Obstetrics & Gynecology Hospital of Fudan University is one of the largest obstetrics & gynecology hospitals in Shanghai, providing both primary and specialty maternal care to more than 10,000 women annually. The hospital is also the first maternal health institution in Shanghai to routinely provide PND screening and mental health services. Mothers are screened for depression at different stages of pregnancy (first, second, and third trimester), before discharge after delivery, and six weeks after delivery, during their routine maternal care. A stepped care model is implemented accordingly as follows.

- EPDS score < 9: The maternal care provider will provide health education on mental health issues and inform the mother of the relevant resources.
- 9 ≤ EPDS score < 13: The maternal care provider will recommend the mother to schedule the on-site outpatient counseling services provided by the hospital.
- EPDS score ≥ 13: The maternal care provider will record the EPDS score in the outpatient health record and discuss treatment options with the mother. If in need, the mother will receive further on-site mental health services through a multidisciplinary team (MDT) approach. Alternatively, she may receive off-site mental health services via referral to psychiatric hospitals. A nurse specialized in perinatal mental health will call the mother back within a week and recommend a follow-up visit to the on-site outpatient counseling services if the diagnosis of any mental disorders is not confirmed. Otherwise, if the diagnosis is confirmed, the mother will be managed by a collaborative care team consisting of an obstetrician, a psychiatrist, and a nurse specialized in perinatal mental health.

Few obstetrics & gynecology hospitals or maternal and child healthcare institutions in China have integrated the screening and intervention for PND in routine maternal care. This hospital is selected as the study site to ensure that mothers with mental health needs could access relevant services during the study.

### ② Study population and recruitment

The study population consists of expectant and new mothers (up to six weeks after delivery) who have screened positive for PND (EPDS score ≥ 13). EPDS usually identifies mild depressive symptoms with a threshold of 9 and moderate to severe depressive symptoms with a threshold of 13 [4]. The current study site routinely uses 13 as the threshold to identify patients with possible moderate to severe PND. This group of patients is included as the study population as they may be particularly at risk if they are not treated or monitored for symptoms.

**Fig. 2:**
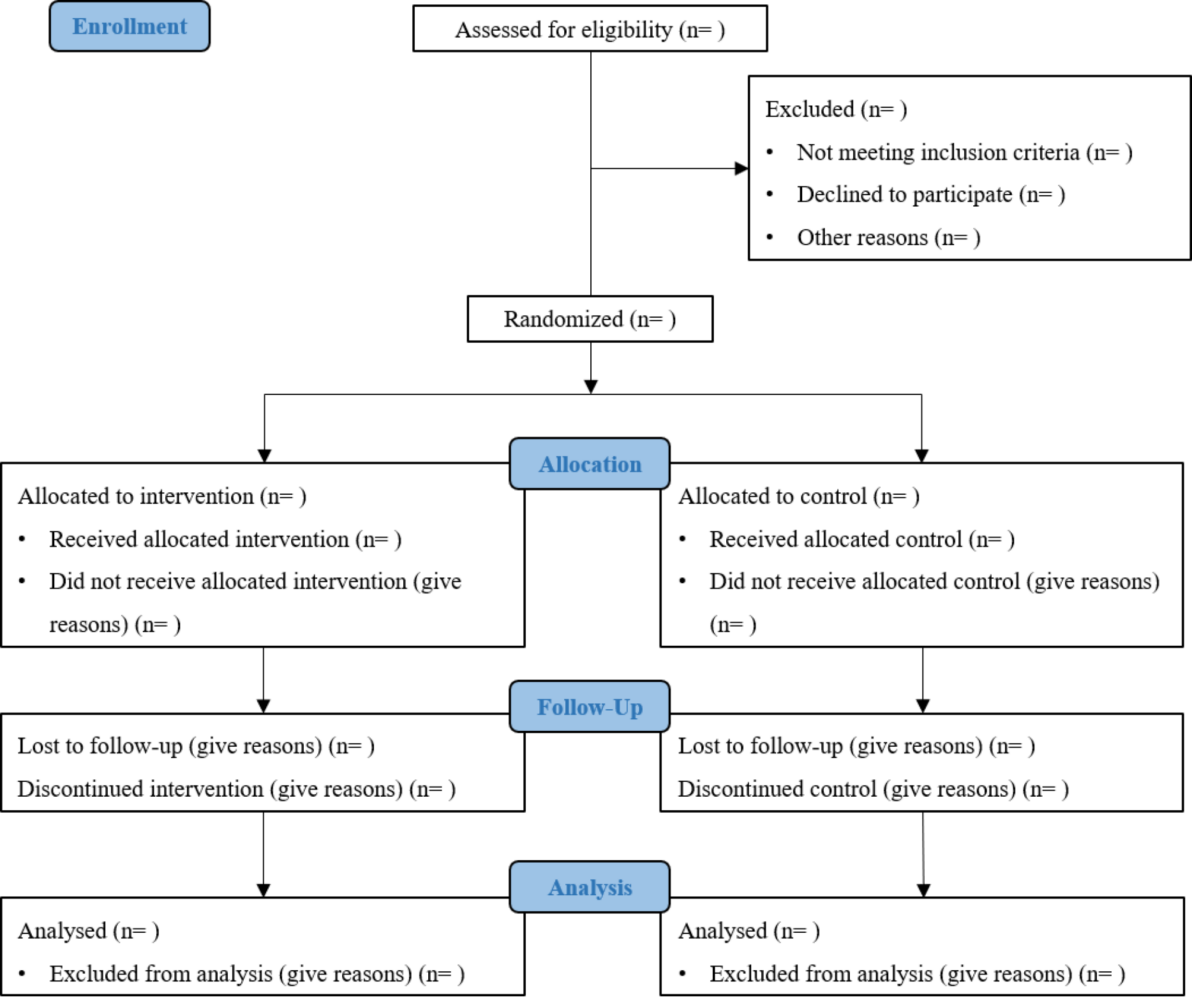
Study flow diagram

The research team will recruit women who screen positive for PND at the outpatient maternal care clinics where women visit for routine maternal checkups. Women who have delivered for more than six weeks are not included as they were only followed up by maternal care providers until six weeks after delivery in routine maternal care. Each mother interested in participating in this study is required to sign an informed consent form. The process of enrollment is shown in Fig. 2.

The Inclusion and exclusion criteria for the study population are as follows.

- Inclusion criteria: 18 years of age or older; a local resident in Shanghai; a registered client at the selected study site to receive maternal health services; and an EPDS score ≥ 13 during pregnancy or within seven weeks postpartum.
- Exclusion criteria: suffering from serious mental illness, namely schizophrenia, delusional disorder, schizoaffective disorder, bipolar disorder, psychotic disorder due to epilepsy, and mental retardation with psychotic symptoms; suffering from other diseases that require immediate hospitalization.

### ③ Randomization and blinding

This study will use permuted block randomization to group the participating women, ensuring a balance across treatment groups. Although a cluster randomized trial (CRT) design would be preferred to avoid potential contamination, simple randomization will be adopted in this single-center study due to the following feasibility issues of conducting a CRT. First, obstetricians at the outpatient maternal care clinics are subject to frequent rotating shifts. Second, there are only several maternal care providers who have been trained to provide on-site mental health services, making them less than ideal targets for randomization and intervention as well.

Prior to recruitment, a non-participating third-party statistician will generate random numbers and create a random assignment table. This table will assign each participant to a group (intervention or control) in a 1:1 ratio based on the order of enrollment. A member of the research team member, who is not involved in participant recruitment, will assign each participant a number in order according to the random assignment table. The assigned number will be placed in a sealed and opaque envelope. During the trial, randomization will take place after a participant has completed informed consent. At the time of opening the envelope, two members of the research team must be present, and the name of the participant with the envelope numbers will be recorded.

Because the intervention group will receive the patient decision aid, and providers will be required to intervene with this group, it will not be possible to blind the mothers and providers. To minimize bias, during the baseline and follow-up assessments, the raters will be blinded to the group assignment.

### ④ Sample size

The primary outcome indicator in this study is the level of decisional conflict at one month after baseline, which is a continuous variable. For the comparison of continuous variable outcome indicators in randomized clinical trials, the sample size can be estimated by the following equation.

Sample size of each group:

$$n = \frac{{2 \times {{({z_{(1 - \alpha /2)}} + {z_\beta })}^2}}}{{{{(\frac{\Delta }{\sigma })}^2}}}$$

Among them, α is set to 0.05, the Z_(1−α/2)_ takes the value of 1.96, while β is set to 0.80, and Z_β_ takes the value of 0.84. ∆/σ is the effect value and is set to 0.3 with reference to previous literature [37], resulting in a required sample size of 174 participants per group. Considering that some of the study participants (assumed to be ≤ 15%) might drop out of the study, the total target of recruiting participants is set at 410 after appropriate expansion of the sample size, and the intervention and control groups are assigned in a 1:1 ratio.

### ⑤ Intervention

Participants in **the control group** will receive stepped care as in routine services (see study site section for details), including PND screening, health education, on-site counseling and MDT services, and off-site specialty referrals.

Participants in the intervention group will receive the IP-SDM care model developed in this study. The IP-SDM care model will provide expectant and new mothers who have screened positive for PND with the patient decision aid, decision coaching, and on-site outpatient counseling services. To establish and implement the IP-SDM care model, the research team will first train nurses specialized in perinatal mental health and obstetricians who provide on-site outpatient counseling services with how to use the patient decision aid to facilitate SDM between patients (including their families) and maternal care and mental health professionals. During the study, the use of the IP-SDM toolkit within the team will be fluid and dependent on the usual roles and processes of care and the needs of the patient. Specifically, participants in **the intervention group** will receive the following interventions in addition to the usual services.

- **Patient Decision Aid**: Participants in the intervention group will receive a patient decision aid for PND prior to their clinical consultation. The patient decision aid will be distributed as a paper booklet, similar in size to the educational materials on perinatal mental health that are routinely distributed at the study site. The patient decision aid will support patients to identify their decision-making problems, understand treatment alternatives, and support them to discuss PND screening results and treatment options with maternal care and mental health professionals, and facilitate SDM.
- **Decision Coaching**: One week after the clinical consultation, the mother will receive a telephone call back from the mental health nurse, during which the nurse will counsel the patient on decision making based on the IP-SDM model. Specific components include identifying decision problems, exchanging evidence-based information, clarifying the patient’s values and preferences, assessing the feasibility of possible treatment modalities, and evaluating preferred options with the patient. The nurse’s decision coaching will also be available to family members if the patient agrees and requires it.
- **Clinical Consultation**: If patients attend the study site’s mental health clinic and the joint psychological specialist clinic, the clinician will also conduct a shared decision-making process with the attending pregnant and postpartum women, following the steps in the IP-SDM model. This process will also engage the family if the patient agrees and requires it.

Mental health nurses at the study site, as well as physicians in the mental health clinic and joint psychiatric specialist clinic, will receive training on the implementation of the IP-SDM model, including how to use decision aids with patients and how to provide decision support to patients in decision coaching and clinical consultation. In addition to the training, the research team will develop communication scripts prepared in a step-by-step decision-making process to help health care providers deliver standardized interventions and thereby prevent “contamination” of interventions.

### ⑥ Measurements

**Fig. 3:**
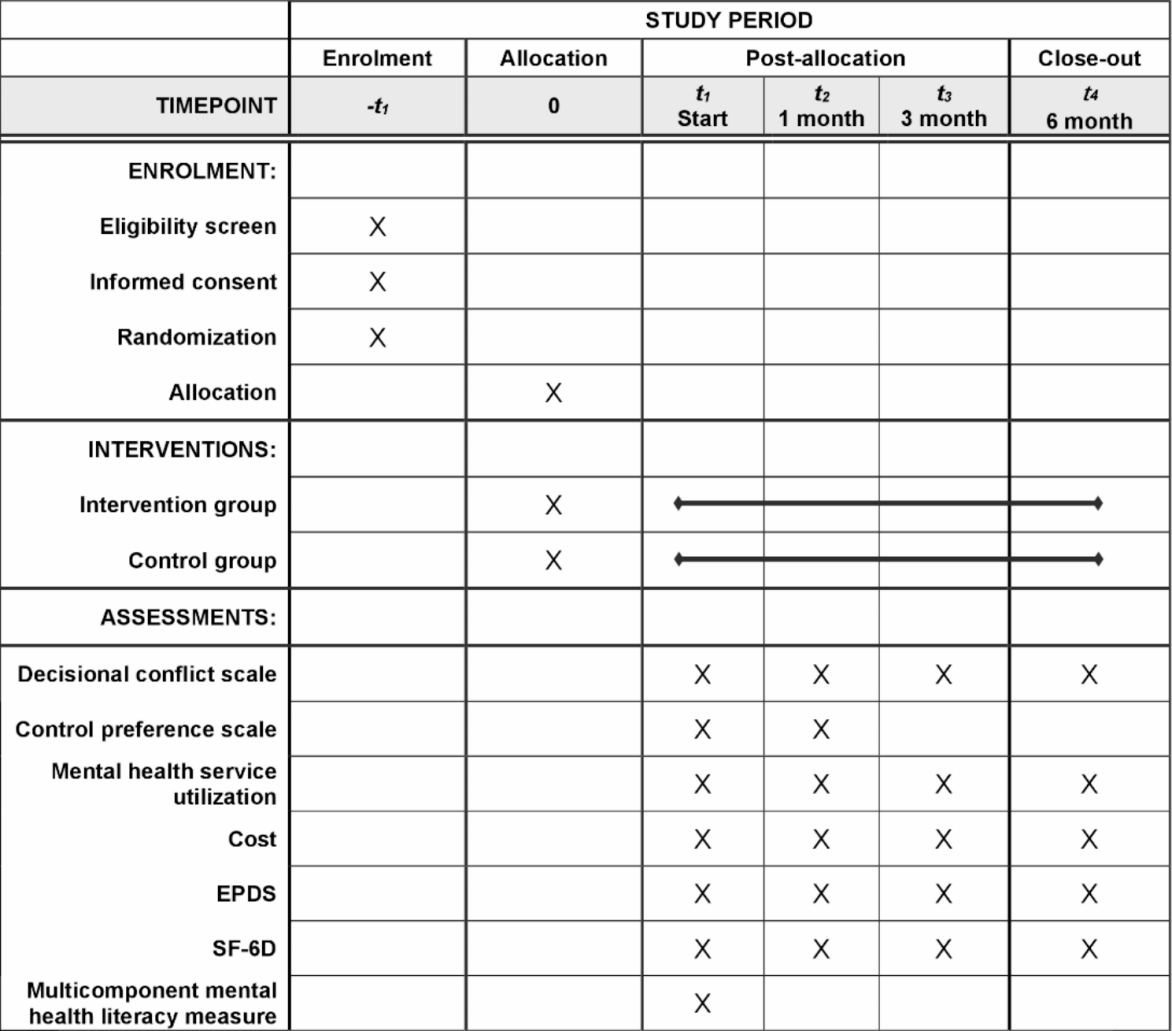
Schedule of enrolment, interventions, and assessments

This trial will observe for a period of six months after baseline. Except for the questionnaire on hospital costs, which will be administered to relevant medical staff and hospital administrators, all other questionnaires will be administered to participants. The baseline survey will be conducted face-to-face, while the follow-up survey will be conducted over the telephone. A face-to-face or online survey can also be arranged if needed. The survey questionnaire will be made into an electronic questionnaire to facilitate completion by either the researcher or the participant. The main indicators (except basic personal information) and measurement methods in each survey are shown in Table 1. The timeline of assessments is shown in Fig. 3.

**Table 1:** Main indicators and measurement methods in previous assessments

Main indicators		Measurement Method
**Primary outcome indicators**		
	Quality of decision-making process	The core indicator of quality of decision-making process is the decisional conflict, i.e. the inner uncertainty of the patient about the treatment option to be chosen during the medical activity. The level of decisional conflict will be measured by the Decisional Conflict Scale (DCS). This scale is the most widely used tool for evaluating decisional conflict and consists of five dimensions: informed, i.e., the extent to which patients understand the potential benefits and risks of the options available to them; value clarification, i.e., the extent to which patients are clear about their values (to measure the benefits and risks); decision support, i.e., the extent to which patients feel supported in their decision; uncertainty, i.e., the extent to which patients are certain about the decision being made; decision validity, which is the patient’s perceived effectiveness, or satisfaction, with the decision. The Chinese version of the scale has also been widely used in studies with good reliability and validity.
**Secondary outcome indicators**		
	Shared decision-making	Shared decision-making will be measured based on an adapted decision expectancy scale (control preference scale, CPS). This scale was originally used to measure patients’ willingness to participate in health care decision-making. In its adaptation, it can also measure patients’ actual participation in decision making in a given decision situation and can classify patients into three types: active decision makers, negative decision makers, and shared decision makers. This is one of the most commonly used and concise tools for measuring shared decision-making.
	Mental health service utilization	A self-administered questionnaire will be used to investigate whether mothers are diagnosed by a psychiatrist with a mental disorder such as PND and the utilization of subsequent mental health services, including all mental health services such as counseling, psychotherapy, and psychiatric services received by mothers at the study site and other institutions or settings (e.g., number of visits, number of counseling sessions, length of medication, etc.).
	Cost	A self-administered questionnaire will be used to measure the costs associated with maternal mental health service seeking. The self-administered scale will be developed with reference to the Treatment Inventory of Costs in Psychiatric Patients and will consist of: direct medical costs, including the costs of maternal psychiatric services, psychotherapy, counseling, etc. (including maternal and health insurance paid costs); direct non-medical costs, including transportation and accommodation fees for maternity and their companions seeking mental health services; indirect costs, including labor losses such as lost wages for maternity and their companions as a result of seeking mental health services. As the related costs are incurred within six months, no discounting is required. Self-administered questionnaires will also be used to measure the cost of conducting the interprofessional shared decision-making model and subsequent mental health services, including mainly depreciation of fixed assets and staff labor costs, among medical staff and hospital administrators.
	Depressive symptoms	Women’s depressive symptoms will be measured by the EPDS, a 10-item maternal self-report of depression over the past 7 days. Higher EPDS scores indicate more severe depression. The EPDS is the most commonly used screening tool for perinatal depression in maternal health care and has good reliability.
	Health-related quality of life	Health-related quality of life will be measured based on the SF-6D, a utility measurement tool created based on the MOS 36-item short-form health survey (SF-36), which is widely used to measure health-related quality of life and can be used to calculate utility values. The SF-6D health classification system consists of 6 dimensions: somatic functioning, role limitation, social functioning, pain, mental health, and vitality. Previous literature has shown that the SF-6D works well in measuring health-related quality of life in mental health and that the SF-6D is more likely to capture the effects of interventions than the EQ-5D in the area of perinatal mental health.
**Control variables**		
	Decision-making preferences	Decision-making preferences will be measured by the Control Preference Scale, which is commonly used to measure patients’ willingness preferences to participate in healthcare decision-making. The scale consists of 5 entries that can classify patients into 3 types: active decision makers, negative decision makers, and shared decision makers.
	Mental health literacy and stigma	The Multicomponent Mental Health Literacy Measure (MMHLM), which is commonly used internationally, is applied in this study to measure maternal mental health literacy and stigma. The 22-item MMHLM, which contains three dimensions of knowledge, beliefs, and resources, has good reliability and validity, is suitable for assessing mental literacy in adult populations, and is also a good predictor of mental health service utilization behavior. The Chinese version of the questionnaire has been tested for reliability and validity in a domestic population.

- **Baseline Survey**: After obtaining informed consent, a member of the research team will conduct a baseline survey. This survey will include basic maternal personal information (e.g., age, education, marital status, family health history, health insurance), level of decisional conflict, history of mental health service utilization, mental health literacy and stigma, and decisional preferences. Among them, basic demographic and health information (e.g., family health history) will be compared with electronic health records for confirmation.
- **Follow-Up Survey**: Follow-up surveys will be conducted at one, three, and six months after baseline. These surveys will include measures of shared decision-making (at one month only), decisional conflict, depressive symptoms, mental health service utilization, and associated costs. The primary outcome of this study is the level of decisional conflict one month after baseline.

### ⑦ Statistical analysis

#### Evaluating the effectiveness of interventions

Quantitative data will be analyzed using STATA according to the principles of intention-to-treat analysis. This study will use t-tests, chi-square tests, and hierarchical generalized linear models to compare the differences in outcomes between the intervention and control groups. For outcome indicators measured at the same time point (e.g., at one month after baseline), t-tests and chi-square tests will be used to compare continuous variables (e.g., level of decisional conflict) and categorical variables (e.g., mental health service utilization), respectively. In contrast, for outcome indicators (e.g., depressive symptoms) that are repeatedly measured at different time points throughout the study period, hierarchical generalized linear models will be used to estimate fixed effects of treatment factors and individual-level random effects.

In addition, this study will use causal mediation analysis to estimate how the levels of SDM affect multiple outcome indicators of patients in the intervention group and the control group, using whether they belong to the intervention or control group as an instrumental variable. Causal mediation effects can be estimated by regression models, depending on the type of exposure, mediator, and outcome variables (whether dichotomous or continuous) and whether the mediating variable interacts with the exposure variable [38]. Analysis of causal mediating effects can also be combined with instrumental variables [39] to analyze the mechanisms by which the levels of SDM itself contribute to multiple patient outcomes.

#### Evaluating the cost-effectiveness of interventions

The cost-effectiveness of implementing the IP-SDM care model will be evaluated over a study period of six months.

From a societal perspective, costs will include those borne by women and health insurance as well as those borne by the hospital. The costs borne by women and health insurance will be estimated based on the predicted values from the regression analysis described above, using data collected from questionnaires. The costs borne by hospitals will be accounted for using the time-driven activity-based costing (TDABC) method [40]. TDABC is a costing method that utilizes the time needed to complete the process to produce a product or deliver a service. In healthcare settings, TDABC is often implemented using seven-step approach, as follows: select the health condition; define the care delivery value chain; develop process maps that include each activity in patient care delivery, and incorporate all direct and indirect capacity-supplying resources; obtain time estimates for activities and resources used; estimate of all direct and indirect resources involved in care delivery; estimate the capacity of each resource and calculate the capacity cost rate (e.g., a cost per unit of time); calculate the total cost of patient care by multiplying the total time of care delivery by the capacity cost rate [41].

In the cost-effectiveness analysis, the effectiveness will be calculated based on the number of women who score 13 or higher on the EPDS at 6 months post-baseline. To compare the cost-effectiveness of between the intervention and control groups, the incremental cost-effectiveness ratio (ICER) will be calculated as the difference in cost between the two groups divided by the difference in their effectiveness.

In addition, the utility will be calculated based on QALYs converted from health-related quality of life as measured by the SF-6D at 6 months post-baseline. ICER will then be calculated as the difference in cost between the intervention and control groups divided by the difference in their QALYs.

### Qualitative evaluation

This study will conduct in-depth interviews among participants, maternal care provider, and hospital administrators. The research team will recruit participants for in-depth interviewees at the study site to explore the facilitating and hindering factors that influence the implementation of SDM.

The inclusion criteria for interviewees will be the same as those for the aforementioned focus group interviews, with the exception that expectant and new mothers should be participants in the intervention group. The exclusion criteria for interviewees will also be the same as those for the focus group interviews.

To protect the privacy of the participants, in-depth interviews are used instead of focus group interviews, as mothers may have prior experience with mental health services at this stage. This study aims to recruit a total of 30 mothers and 15 maternal care providers and hospital administrators in total. However, the recruitment will continue until data saturation is reached. The interviews will last between 0.5 and 1 h. For expectant and new mothers, the interviews will focus on the use of decision aids, as well as their experiences and perceptions related to SDM. For maternal care providers and hospital administrators, interviews will focus on perceived facilitators and barriers to SDM in maternal mental health services. The data analysis methods for the in-depth interviews are the same as those described previously for the focus group interviews.

## Discussion

Previous literature has documented the challenges of implementing SDM in the real-world settings. Some providers are concerned about the strength of evidence supporting decision aids, increased workloads associated with using them, and the lack of supportive environment [42]. Patients may find it challenging to comprehend the information presented in decision aids, and they may not want decision-making responsibility when facing difficult diagnoses [43]. To address the implementation challenges of incorporating a patient decision aid into clinical practice, this study aims to develop and evaluate an IP-SDM model for patients with PND in maternal care. The IP-SDM model will encompass a patient decision aid as its centerpiece, as well as multiple intervention components such as decision coaching and clinical consultation delivered by a multi-disciplinary team of providers. The proposed IP-SDM model will involve task shifting and sharing to alleviate the workload of providers. For instance, decision coaching will be conducted by nurses. Considering the limited time frame of maternal care consultations in China’s public hospitals, nurses may be better suited to communicate accurate and up-to-date evidence to patients and make decisions with patients based on their needs and preferences. Also, this study will adopt a participatory design approach to ensure that the patient decision aid can be understood by expectant and new mothers. The IP-SDM model may engage the family as well if the patient wants to share the decision-making responsibility with her family.

Furthermore, the IP-SDM model aims to increase acceptance of evidence-based mental health services for PND in maternal care settings. In these settings as well as other primary care settings, patients may not seek care for mental health concerns due to stigma or lack of mental health literacy [44]. Additionally, providers may lack the necessary skill sets or time to deliver mental health services [45]. Obstacles at the health system level may include a lack of political commitment, resources, guidelines, and health information systems to facilitate collaborative care [46–48]. To address these potential challenges, this study will develop a patient decision aid based on the best available evidence that can support both patients and providers in the decision-making process. In addition, with input from various stakeholders including hospital administrators, the IP-SDM care model will be integrated into the clinical practice to overcome potential organizational obstacles. Also, the cost-effectiveness of implementing the IP-SDM model will be evaluated to facilitate its future dissemination.

This study has several limitations. First, contamination can be a threat that biases the results towards null. Contamination is possible since it is not feasible to conduct a CRT design as discussed before. To identify the effects of SDM and alleviate the bias that may be introduced by contamination, this study will use randomization as the instrumental variable and examine how the assessed level of SDM after intervention would affect outcomes of interests. Second, the generalizability of this study should be interpreted with caution. The study site of this study was selected to ensure that mothers with mental health needs could access relevant services during the study. However, only a few maternal and child healthcare institutions in China have already integrated the screening and intervention for PND in routine maternal care. Thus, the findings may not be generalized to other regions of China. To maximize generalizability, this is an open-label trial with its interventions fully embedded into daily clinical practice.

Despite these limitations, this study will make the following contributions to the field. First, this study aims to contribute to our understanding of how SDM impacts mental health outcomes. This study will investigate not only how SDM affects mental health outcomes but also explore the potential mechanisms underlying such impacts, using causal mediation analysis. Second, this study will develop a culturally relevant and responsive IP-SDM model for the Chinese population. Most previous studies on SDM were conducted in high-incomes countries, with a lack of research on the Chinese population. Through a participatory design approach, the IP-SDM model will take into account the perspectives and cultural contexts of various stakeholders. For instance, the IP-SDM model will incorporate the input of family members in the decision-making process between patients and providers. Lastly, this study will explore an IP-SDM model to integrate mental health services into maternal care. The findings on the role of SDM in the diagnosis and treatment of PND could have important implications for other common mental disorders in the general population.

### Electronic supplementary material

Below is the link to the electronic supplementary material.

Supplementary Material 1

Supplementary Material 2

## Data Availability

Data sharing is not applicable to this study protocol.

## Abbreviations

PND: Perinatal depression
SDM: Shared decision-making
IP-SDM: Interprofessional shared decision-making
RCT: Randomized controlled trial
SPIRIT: Standard Protocol Items:Recommendations for Interventional Trials
IPDAS: International Patient Decision Aids Standards
CTA: Cognitive task analysis
EPDS: Edinburgh Postnatal Depression Scale
OPTION: Observing Patient Involvement in Decision Making
MDT: Multidisciplinary team
CRT: Cluster randomized trial
TDABC: Time-driven activity-based costing
ICER: Incremental Cost-Effectiveness Ratio
