# Supplementary material for: Developing and evaluating an interprofessional shared decision-making care model for patients with perinatal depression in maternal care in urban China: a study protocol
Source: BMC Prim Care. 2023 Nov 3;24:230. doi: 10.1186/s12875-023-02179-2 (PMC10623702; doi:10.1186/s12875-023-02179-2)
Supplement: Supplementary file 2 — Supplementary Material 2 [file 12875_2023_2179_MOESM2_ESM.docx]

**Focus group interview questions for expectant and new mothers**

1. What do you think of perinatal depression?
2. Do you know anything about treatment modalities for perinatal depression? Do you have any expectations or preferences for choosing these modalities?
3. What support and resources would you need if you want to make treatment choices for perinatal depression?
4. What do you think of the decision aid we prepared?

**Focus group interview questions for obstetric professionals**

1. What is your perspective on perinatal depression and its treatment modality?
2. Do you have any expectations or preferences for these treatment modalities? What expectations and preferences do patients have from your perspective?
3. What support and resources would you need if you want to make treatment choices for perinatal depression with patients?
4. What do you think of the decision aid we prepared?
